# The Modern Workhouse Infirmary

**Published:** 1886-11-20

**Authors:** 


### The Modern Workhouse Infirmary.

The Bad Old Times.

In a recent article I gave some account of the old-fashioned workhouse infirmary. It was but a partial and modified account, because there were in connection with the workhouses of a quarter of a century ago details of management so disgusting, and incidents of workhouse invalid life so painful and repulsive, that, without some special and imperative purpose, it would hardly be justifiable to rake them up and set them forth in print. From those closing days of the old *régime* of Bumbledom it is pleasant to turn to the new order of things as illustrated in the best modern Infirmaries. In doing this let all honour be given to those who have brought about these much needed reforms. The names of Anstie, Ernest Hart, Miss Louisa Twining, the Editor of the *Lancet* and his able staff, deserve to be held in grateful remembrance by us all, for to them must be credited the results and improvements at which all must rejoice, and to which we will now direct attention.

The New Times too Good.

Upon such an establishment as that opened for the benefit of the poor of St. Marylebone in 1881, only one point of criticism will be found, by the ordinary visitor at any rate. The only fault it will be possible to find is that it is too good. The situation, the building, the adornment of the wards, the medical attendance, the beds, the nursing, the food—all are of a character that could not be materially improved upon. Things might no doubt be more sumptuous and showy, but in none of the things really essential to the recovery of health and to the comfort of the patients could money do much more than the St. Marylebone guardians have done here; and the ratepayer who goes through these spacious corridors and airy wards, in which bright pictures look down on snow-white beds, and the cheerful beams of the sun play among groups of flowers and foliage clustered here and there about the floors—who looks out upon pleasant lawns and open fields, peeps into bath-rooms such as his own money has never been able to provide for his own family, and sniffs at kitchen odours that would do no discredit to a first-class hotel, is very apt to feel that parochial benevolence has been a little overdrawn, and that if guardians of the old school had too great a regard for the ratepayers' money, surely the new school has somewhat too little.

Not too Good After All.

Now we must be on our guard against sentimentalism. To be sick and suffering as well as destitute must always constitute a strong claim upon our sympathies, even though indiscretions and weaknesses, follies and vices may have done much to bring about this sickness and suffering. But however much we may sympathise with the destitute in time of illness, it would be obviously unfair to indulge them in needless luxuries to be paid for by ratepayers, many of whom, while they have none of their vices and follies, have a battle of their own to fight as severe as that in which the pauper has gone down. Nothing approaching extravagance ought to be tolerated in these places. One's first impression of the best of the workhouse infirmaries is that they certainly are too good. Examination in detail, however, fails to discover a single item on which a charge of extravagance could be sustained. Certainly, this St. Marylebone Infirmary is a finer range of buildings than it need be; that is to say, it might have been plainer and cheaper without materially affecting the welfare of the inmates. Something, however, is due to a neighbourhood in which huge institutions of this kind are erected, and no doubt it was this consideration which induced the guardians of St. Marylebone to put up what is really a very fine institution architecturally.

The Infirmary stands at the bottom of

The Marylebone Infirmary Described.

Rackham Street, Notting Hill. An entrance-gate leads into a private road. Going down this road, there is on the left-hand side a "Home" for the training of nurses in connection with the infirmary. This is under the direction of the "Nightingale" as well as the Infirmary Committee, with Miss Vincent as lady superintendent, and is the first of the kind attached to a Poor-law hospital. On the right-hand side of the road lies the main building. Entrance to it is under a Gothic archway in a block comprising the residence of the medical superintendent, the lady superintendent, the assistant medical officer, and a chapel with accommodation for about 250 people. Separated from this entrance-block by an open space is a larger block, in which are the administrative offices, with engineers' shop, furnace, and boiler rooms in rear. Adjacent is a large and handsome tower with a furnace-chimney in the centre, around which winds a staircase leading to the laundry, washhouse, and drying space situated in the upper storeys. The infirmary proper consists of four pavilions, two on each side of this central range of buildings, with pleasant open spaces between them, the whole of the four pavilions being united with the central administrative offices by corridors 10 feet wide, running out right and left. These pavilions are three storeys high, and on each floor they have two wards 84 feet long, 24 feet wide, and 13 feet high. The wards have a particularly bright, pleasant, airy look about them. There are windows on both sides; the walls are lightly coloured, with a dark dado on the lower part; coloured prints and illuminated texts —the gifts of charitably-disposed visitors—lend a cheerful and well-furnished aspect to the place; and the flowers and ferns already alluded to—like the pictures, provided without cost to the ratepayers—combine with the whiteness of the beds and the scrupulous cleanliness of every detail to present a scene contrasting in the most striking way with the old-fashioned workhouse infirmary ward. There are on the various floors day-rooms for patients, small wards for the separation of special cases, and nurses' duty rooms. There are also lavatories and water-closets and bath rooms at the outer ends of the wards; and it is significant of the scrupulous care with which every detail of this model establishment has been worked out, that these laundries and so forth are cut off from the wards by an intervening small lobby, with an open window on each side; and lest, even with this precaution, any unwholesome emanations should find their way to the patients, it has been arranged that all these offices shall be kept at a rather higher temperature than that of the wards, so that there may always be a slight current of air from the wards through the ventilating lobbies rather than in the contrary direction. After this it need not be said that the ventilation of the wards generally has been most carefully provided for. The whole building is lighted by gas, with provision for the entire exclusion of the products of combustion. The water is supplied by an artesian well; the drainage is the best, and fire-mains and hydraulic lifts are provided throughout the building.

A Perfect Institution.

It is difficult to conceive of any great improvement in this as an institution for the sick poor in any respect whatever. The whole place occupies a little over four acres, of which the actual buildings cover about an acre and a half, so that with roads, gardens, and yards, about a third of the whole site is open space. The guardians of St. Marylebone have, in fact, set up a model infirmary. With that comprehensive and far-sighted wisdom which it is to be hoped will become more and more characteristic of poor-law guardians, they have judged that, upon the ground both of humanity and economy, it is expedient to do all that can be done—not to keep such people on their hands, or to let them die and leave their kith and kin burdens upon the parish rates—but to cure them as speedily as possible. A just tribute must be awarded to Miss Vincent, who has proved herself to be one of the ablest Lady Superintendents in the country. To her capacity, tact, sound judgment, and exceptional administrative ability, the efficiency of the Marylebone Infirmary is largely to be attributed; and her selection for the post she now holds was undeniable proof of the wisdom of the Guardians of Marylebone.

Not Quite Perfect in every Respect.

In one respect, to which I may have to revert on another occasion, they have not yet quite the courage of their opinions—and it is a very important respect too—but they have at least set up a splendid hospital in a fine, healthy, open situation. They have secured abundant light and pure air and good water, cheerfulness and comfort, and good nursing and medical skill, and though the place looks a costly one, the architects, Messrs. Snell and Son, maintain that it is in fact an exceedingly economical one. The number of sick for which accommodation is provided is 744. The resident officers and servants employed by the institution is 86; and the total cost, including land, buildings, and all sorts of fittings and professional fees, was £143,000. This is at the rate of £193 per bed. But the architects quote corresponding figures for twelve hospitals erected on the same principle—the isolated pavilion principle, that is—during the past twenty years or so, and this, on the score of economy, compares very favourably with all of them. Messrs. Snell state that one at Blackburn cost £307 per bed; one at Leeds £326; at Genoa an infirmary was put up at a cost of £794 per bed; St. Thomas's cost at the rate of £967 per bed; and the new Hotel

Dieu, in Paris, erected during the years 1866-76, in place of the dreadful old institution referred to in a previous article, cost, it is said, no less than £2,487 per bed for land and buildings.

Twenty Years Ago.

Only twenty years ago or thereabouts competent witnesses declared of one of the best of London workhouse infirmaries, that the general aspect of its wards was one of extreme cheerlessness and desolation. This, we are told, was painful throughout, but especially lamentable in the case of the lunatics and imbeciles, at that time very commonly kept for a long while at the parish infirmary. Moping about in herds, without any occupation whatever, neither classified nor amused nor employed, congregated in miserable day-rooms, where they sat and stared at each other or at the bare walls, when the monotony was not broken by the occasional excitement due to an epileptic or the gibbering and fitful laughter of some excitable lunatic, the case of these poor mortals was pitiable indeed; and when we add to this all the other distressing features of old-fashioned workhouse treatment of the sick, previously described, we cease to wonder that to this day—such is the horror the mere tradition of these places has inspired in the poor—that they often prefer to die in their own wretched garrets rather than go into "the house."

To-day.

Bearing in mind these things, one leaves an institution like this St. Marylebone Infirmary with a profound sense of thankfulness. Here, at least, the light of science and common sense and divine pity has flooded out the old brutality and ignorance, and one feels encouraged to hope that the time is not far distant when all the still dark corners of sick pauperism will be similarly flooded.

